# Treatment of Fractures of the Thigh Bone

**Published:** 1829

**Authors:** 


### II Analytical.

25. *Treatment of Fractures of the Thigh Bone.*—In the Am. Jour. for Aug. 1829, Dr. Daniell of Savannah, has added one more to the numerous publications on this subject. His fundamental proposition is to effect extension by means of a weight and cord passing over a pully from the foot. We shall transcribe that part of his short paper which describes his method.

With the assistance of my friend Dr. Richardson, I have recently treated a case of oblique fracture of the thigh bone after the following manner:—A piece of poplar plank, long enough to extend from just below the buttock to eighteen inches beyond the foot was made on the surface slightly concave to receive the thigh—the upper end was cut into a semilunar form to fit it the better to the buttock, and made six inches wide—the lower end was four inches wide. On each side of the lower end was attached a piece of board three in-

*Clinique, No. 5. Tome 4.

ches high, extending up to the knee, with a gradually reduced height. A piece of board five inches high, was then fitted in the lower end, at a right angle with the lower board. In the middle of the upper edge of this piece was placed a small wooden roller, with a cancave edge, which was retained by a wire axis. The lower end of this splint, which projected beyond the foot of the bed, was secured by passing a screw through the bottom piece into the foot-board of the bed. The fractured limb was then placed in this splint. The many-tailed bandage was applied over the fractured portion, (the bones having first been placed in apposition,) over which at equal distances a part and round the limb, four thin wooden splints, six inches long, were placed and secured by muslin strips. A bag of dried moss was then applied on each side of the thigh, and secured by tapes passing under the board supporting the thigh, and over the limb. A silk handkerchief was then passed around the ankle, and tied at the bottom of the foot. To this projecting portion of the handkerchief was fastened a small flaxen cord, and that passing over the roller placed in the end of the case, supported a small weight. A muslin bandage was passed around the chest to which bandages were fastened for the purpose of fixing the body to the head-board to prevent its being drawn down. This was however soon found to be superfluous, as the weight of the body was quite sufficient for the purpose of resistence to the extending power, and was consequently discontinued. The dressings were renewed once or twice a week, according to circumstances, and the bones united readily, and without any shortening of the limb.

That portion of our patient's mattrass which supported the breech was made removeable, by which arrangement the pan could be used without inconvenience.

Whenever any shortening of the fractured limb was observed, the leg was gently raised and extended to the proper distance, where it was retained by the weight attached to the cord. And here I will observe, that the cord and weight are rather designed for retaining the limb properly extended than for extending it. The latter it is known is readily performed. The importance as well as the difficulty of keeping up that extension, has been felt by every surgeon who has had a fractured thigh to treat. I flatter myself that the above mode of making and maintaining the extension, will be found an improvement. It has certainly been such in my hands.—pp. 331–2.

Dr. Daniell thinks that in general, a weight of two pounds will be sufficient, which may be the case, after the muscles have become fatigued, and ceased to contract with energy, but would we suppose be too little in the beginning.

In the conclusion of his paper, Dr. D. enters a protest against placing a fractured thigh in complicated dressings, immediately after the accident. We have already in a former number expressed ourselves against this practice, and are happy to find that others entertain the same opinion.

